# A simple and efficient method for isolating polymorphic microsatellites from cDNA

**DOI:** 10.1186/1471-2164-10-125

**Published:** 2009-03-25

**Authors:** Gen Hua Yue, Ze Yuan Zhu, Chun Ming Wang, Jun Hong Xia

**Affiliations:** 1 Molecular Population Genetics Group, Temasek Life Sciences Lab, 1 Research Link, National University of Singapore, 117604 Singapore

## Abstract

**Background:**

Microsatellites in cDNA are useful as molecular markers because they represent transcribed genes and can be used as anchor markers for linkage and comparative mapping, as well as for studying genome evolution. Microsatellites in cDNA can be detected in existing ESTs by data mining. However, in most fish species, no ESTs are available or the number of ESTs is limited, although fishes represent half of the vertebrates on the earth. We developed a simple and efficient method for isolation of microsatellites from cDNA in fish.

**Results:**

The method included normalization of 150 ng cDNA using 0.5 U duplex-specific nuclease (DSN) at 65°C for 30 min, enrichment of microsatellites using biotinylated oligonucleotides and magnetic field, and directional cloning of cDNA into a vector. We tested this method to enrich CA- and GA-microsatellites from cDNA of Asian seabass, and demonstrated that enrichment of microsatellites from normalized cDNA could increased the efficiency of microsatellite isolation over 30 times as compared to direct sequencing of clones from cDNA libraries. One hundred and thirty-nine (36.2%) out of 384 clones from normalized cDNA contained microsatellites. Unique microsatellite sequences accounted for 23.6% (91/384) of sequenced clones. Sixty microsatellites isolated from cDNA were characterized, and 41 were polymorphic. The average allele number of the 41 microsatellites was 4.85 ± 0.54, while the expected heterozygosity was 0.56 ± 0.03. All the isolated microsatellites inherited in a Mendelian pattern.

**Conclusion:**

Normalization of cDNA substantially increased the efficiency of enrichment of microsatellites from cDNA. The described method for isolation of microsatellites from cDNA has the potential to be applied to a wide range of fish species. The microsatellites isolated from cDNA could be useful for linkage and comparative mapping, as well as for studying genome evolution.

## Background

Microsatellites are short segments of DNA in which a specific motif of 1–6 bases is repeated [1,2]. Due to their high polymorphism, codominant inheritance, ease of scoring and dense distribution throughout eukaryotic genomes, microsatellites are now generally considered to be the most powerful genetic markers for genetic mapping and evolutionary studies [3]. One perceived difficulty with microsatellites is the long lead time in identifying and characterizing microsatellites in new taxonomic groups. This problem is alleviated by developing novel protocols for enriching repeat DNA from genomic DNA [4]. However, most microsatellites are type II markers for which no known function has been established. Type I markers are associated with genes of known functions and are more useful for comparative gene mapping to study genome evolution [5] and for identifying markers associated with important quantitative traits [6]. Although SNPs in genes were identified in some fish species [7,8], type I markers are still relatively rare in fish. Detection of polymorphic microsatellites located within transcribed genes provides a possibility to convert type II markers to type I markers [9]. Previous studies demonstrated that some microsatellites with genes were associated with economically important traits [6,10], and could be used as markers for marker-assisted selection. Currently, microsatellites in transcribed genes have been identified in model organisms [11] and economically important animal [8,9,12-14] and plant species [15,16] by data mining in ESTs using bioinformatics tools or direct sequencing ESTs. However, in most of 31,000 fish species existing on the earth [17], it is difficult to obtain microsatellites in cDNA through data mining, due to the fact that no ESTs are available, or the number of ESTs is limited in these species. Although a method for enriching microsatellites from genomic DNA has been adapted to identify microsatellites from cDNA in catfish [18], the efficiency of isolation of microsatellites in cDNA is still not very high as comparing that in genomic DNA [19], due to the redundancy of cDNA. In this paper, we report a very simple and efficient method for isolating microsatellites from transcribed genes. The method included cDNA normalization, microsatellite enrichment and directional cloning of cDNA enriched with microsatellites.

## Results and discussion

In a previous study [10], we sequenced 4800 ESTs from six normalized cDNA libraries of Asian seabass (*Lates calcarifer*). From the 4800 ESTs, a total of 70 unique sequences containing microsatellites (repeat length: dinucleotide > 7, trinucleotide > 6, tetranucleotide > 5) from 130 clones were identified. Among the 70 microsatellites, 42 were CA-repeats, 23 GA-repeats, two GGA-repeats and three other types of repeats. These data indicate that unique microsatellite sequences accounted for 1.45% (70/4800) of cDNA clones in Asian seabass. CA- and GA-microsatellites were most abundant in cDNA of Asian seabass. However, they represent only 0.83% (40/4800) and 0.48% (23/4800) of cDNA clones from normalized cDNA libraries. Hence, straightforward random sequencing of clones from normalized cDNA libraries is not efficient for discovering microsatellites.

In this study, we tried to enrich CA-, GA-microsatellites from unnormalized cDNA of Asian seabass using biotinylated (CA)_10 _and (GA)_10 _oligonucleotides, since these two types of microsatellites are most abundant in cDNA of Asian seabass. Two cDNA libraries were constructed, one enriched for CA-microsatellites and another for GA-microsatellites. From each library, 192 randomly picked clones were sequenced in both directions. Among the 192 clones from the cDNA library enriched for CA-repeats, 80 clones contained microsatellites. Of the 80 clones containing microsatellites, only 11 were singletons, and the remaining 69 were included in 8 clusters. A total of 19 (9.9%) unique microsatellites were obtained from the 192 sequences clones (Table 1). Similarly, among the 192 sequenced clones from the cDNA library enriched for GA-repeats, 40 clones contained microsatellites. Eight were singletons, and 32 were included in 6 clusters. The cDNA sequence of the parvalbumin gene beta-1 containing one CT-microsatellites [10] appeared 8 times in the 192 clones. A total of 14 (7.3%) unique microsatellites were obtained from the cDNA library enriched for GA-microsatellites. In comparison to the random sequencing of clones from normalized cDNA libraries without enrichment of microsatellites, the efficiency of microsatellite isolation from unnormalized cDNA libraries enriched for microsatellites has been raised over 10 times (for CA microsatellites: 9.9% vs. 0.83%; for GA-microsatellites: 7.3% vs.0.43%). In catfish, similar efficiency of isolation of microsatellites from cDNA was reported [18]. However, high redundancy of cDNA sequences from unnormalized cDNA libraries reduced the efficiency of microsatellite isolation from cDNA.

**Table 1:** Comparison of the efficiency of microsatellite enrichment from unnormalized and normalized cDNA libraries of Asian seabass

cDNA	Enriched for	No clones ^a^	No MS clones^b^	No singlteons ^c^	No clusters ^d^	No unique microsatellites	%
Unnormalized	(CA)n	192	80	11	8	19	9.9
	(GA)n	192	40	9	5	14	7.3
Normalized	(CA)n	192	88	41	10	51	26.5
	(GA)n	192	51	35	5	40	20.8

^a^: Number of clones sequenced; ^b^: Number of clones containing microsatellites; ^c^: Number of singletons containing microsatellites; ^d^: number of clusters containing microsatellites, and %: percentage of clones containing unique microsatellites

**Figure 1:**
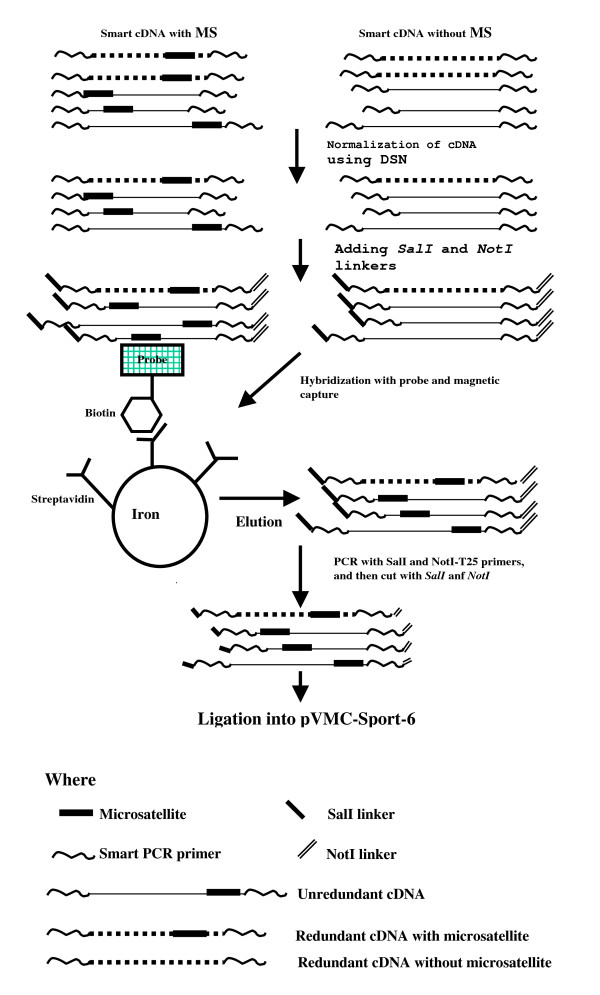
**Schematic presentation of the method for microsatellite enrichment from normalized cDNA**. Details of each step can be found in the section "Methods".

**Figure 2:**
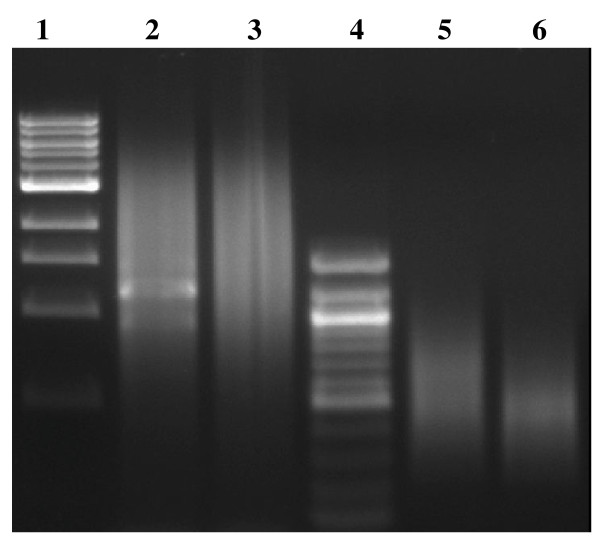
**Agarose gel electrophoresis (1%) of smart cDNA, normalized cDNA and cDNA enriched with microsatellites**. Lane 1: 1 Kb ladder (NEB); Lane 2: smart cDNA; Lane 3: normalized cDNA; lane 4: 100 bp ladder (NEB); lane 5: normalized cDNA enriched for CA-microsatellites and lane 6: normalized cDNA enriched for GA-microsatellites.

In order to increase the efficiency of enrichment of microsatellites, we tried to reduce the redundancy of cDNA by normalizing cDNA using duplex-specific nuclease (DSN) [20] before enrichment of CA- and GA-microsatellites (Figure 1). After cDNA normalization, redundant cDNA were removed (Figure 2). Two normalized cDNA libraries, one enriched for CA-microsatellites and another for GA-repeats were created. From each library, 192 clones were sequenced in both ends respectively. Eighty-eight (45.8%) and 51 (26.5%) clones of the 192 clones from the normalized cDNA libraries enriched for CA- and GA-repeats respectively, contained microsatellites (Table 1). The redundancy of clones was substantially reduced. In the 88 clones containing microsatellites from the cDNA library enriched for CA-microsatellites, 41 were singletons, the remaining 47 were included in 10 contigs. A total of 51 (26.5%) unique microsatellites were obtained from 192 sequenced clones. In the 51 clones containing microsatellites from the cDNA library enriched for GA-repeats, 35 were singletons, and 16 were included in 5 clusters. A total of 40 (20.8%) unique microsatellites we obtained from 192 sequenced clones (Table 1). In comparison to the efficiency of microsatellite enrichment from unnormalized cDNA, the efficiency was about three folds increased by using normalized cDNA (for CA enrichment: 26.5% vs. 9.9%; for GA enrichment: 20.8% vs. 7.3%). Therefore, decreasing the prevalence of clones representing abundant transcripts before microsatellite enrichment by normalization of cDNA is essential for microsatellite isolation from cDNA. The normalization of cDNA using DSN was very simple and highly efficient in comparison to other cDNA normalization methods [21]. The whole procedure of microsatellite enrichment starting from normalization of cDNA lasted only 5 days. Application of this method to isolate microsatellites from cDNA of grass carp brain got similar results (data no shown). Therefore the method is robust and reproducible.

Sixty of 91 microsatellites isolated from the libraries enriched for CA- and GA-microsatellites had enough flanking regions for designing primers, and were characterized in a panel of 24 individuals previously used for characterization of microsatellites isolated from genomic DNA [22]. Forty-one were polymorphic with an average allele number of 4.85 ± 0.54 ranging from 2 to 20 (Table 2), whereas the average expected and observed heterozygosity were 0.56 ± 0.03 and 0.47 ± 0.04 respectively. The average allele number of microsatellites isolated from cDNA is slightly lower than those isolated from genomic DNA libraries, and characterized with same DNA panel [22], which might be due to the relatively lower number of repeats of microsatellites identified from cDNA. Examination of genotyping errors using MicroChecker revealed no evidence for large-allele dropout or stutter-band scoring at any of the 41 loci. All 41 polymorphic microsatellites showed a Mendelian pattern of inheritance. Twenty-nine of 41 microsatellites were in HWE (Table 2). Departure from HWE at 12 loci may be caused by the presence of null alleles. However, examination of genotypes using MicroChecker showed the possibility of presence of null alleles is low (*P *> 0.05). Therefore, microsatellites isolated from cDNA using the described method could be useful for linkage mapping and comparative mapping and studies on genome evolution.

**Table 2:** Characterization of 41 microsatellites isolated from cDNA of Asian seabass

Locus GenBank No	Repeat motif	Primer sequence (5'-3')	Allele number	Size range (bp)	*Ho*	*He*
*LcaE072*	(TG)_18_	CGCGGGGTTCCTCTTTCA	4	196–202	0.17	0.33*
EF210110		GCACCTGCACAGCTACACTCT				
*LcaE073*	(CA)_8_	CGGGTAGAAGACTGGAGGAGAG	9	298–338	0.71	0.79
EF210111		ACATTTTGCCACCATTTTGACTT				
*LcaE74*	(TG)_9_	TCTGGGGGCTGGTATATTTTCCTG	2	396–398	0.42	0.45
EF210112		ACACATTCCACCGCTGCCATTT				
*LcaE075*	(TG)_11_	CGCGGGATTGCCACTTTATTTT	5	226–234	0.38	0.56*
EF210113		GAGGATGGACTTCACAGGCACAGA				
*LcaE076*	(TG)_28_	GGGATCAGAACAAGAAAACAACC	20	117–185	0.91	0.92
EF210114		TAATCTAGGCACCAGGCTTCTTC				
*LcaE077*	(TG)_7_	CCTCACTGTGTCCAAGCATC	2	166–168	0.54	0.51
EF210115		TCCCCCTCAATAAGTAAAAATAG				
*LcaE079*	(CA)_13_	CCCGCCAAGGTCTCGTGTC	3	186–196	0.25	0.27
EF210116		CGGCAGCTGCGTGAATGTG				
*LcaE080*	(GA)_12_	GTCGCACCAGGTTAGGAGA	3	142–146	0.35	0.66*
EF210117		AAAAAGAGGAGCCACAGAGA				
*LcaE201*	(AG)_13_	GGCAAGCAGCCTCGGAGAAA	7	140–158	0.83	0.66
EF210118		TAACGGTTTTGGAAATTGAGATGC				
*LcaE203*	(CT)_14_	AGGCCCGAGGGTGAGCAG	6	262–278	0.54	0.83*
EF210119		ATGCAAAGGTGCGTGTATGATTTT				
*LcaE205*	(CA)_12_	CTGTGTCGCCTGTCGTAGTGACTG	6	195–213	0.46	0.61*
EF210120		CATTTGCTCCCATACTGGAACTGC				
*LcaE206*	(CA)_8_	GAAGCGGTCGAGGAAGGAATCC	3	132–136	0.41	0.46
EF210121		AGGCTTGGTACTTGCAGTGCTGTG				
*LcaE208*	(AC)_10_	GTCAGGCTGGCGACCATCTCA	3	176–180	0.44	0.46
EF210122		CAAATTCAAACCTTTATTAACACAACTA				
*LcaE209*	(AC)_16_	GTGTGAAACCAGGGAGCGTATG	7	208–240	0.58	0.77
EF210123		CCTTTGGGCAGTACTGTATTTTTC				
*LcaE210*	(TG)_10_	TCTGTACAAGTGGCACTCCATCAT	5	70–78	0.38	0.57*
EF210124		CAATCAAGCTTCACTGTTTTTACC				
*LcaE211*	(TG)_20_	TTTTTGAGGGGTACAATAAGTGTG	13	202–278	0.58	0.91*
EF210125		GAGAGCGGGATGAAGTATGAAG				
*LcaE014*	(TC)_11_	TGGAGAATGACACAAACGAGTAAA	6	256–266	0.58	0.76
FJ535708		ATTTGGGTGCATAGTGAGACAGTC				
*LcaE015*	(CA)_16_	AAGGCAAAGAGACACACACAC	5	153–167	0.68	0.66
FJ535709		CTCGATTCACTCTGCTCCATG				
*LcaE016*	(TC)_8_	AGCAGCGGAGGTGGAGTCA	4	258–266	0.87	0.66*
FJ535710		GGAGGGAGGGAGGGAGGAG				
*LcaE020*	(TG)_10_	ACCCCACGCAAGGAGAGGATAAT	3	191–195	0.35	0.36
FJ535711		GCTGGCAGGCGTTGTAATGAGTT				
*LcaE021*	(CA)_7_	CCTCAGTAATTTGGTCAAGAAACT	3	144–158	0.44	0.33
FJ535712		TGCAAGGACCTCTATGATACAAA				
*LcaE022*	(GA)_10_	ACTAACCCCGTTTGGCGTCCATCT	2	304–306	0.44	0.42
FJ535713		TTTGGCTTCGTTGTGATCATCAGC				
*LcaE082*	(TC)_5_–(TG)_8_	ATGGGGGTGGGGGTGTAACTTT	2	326–328	0.51	0.50
FJ535714		GCAGGGGCCCGGCTCATA				
*LcaE083*	(TG)_23_	GTCAAGGTAAACTGCCAATAG	3	154–158	0.47	0.38
FJ535715		AGAGAAAATGAGAGGAAGTTAGG				
*LcaE098*	(CA)_12_	GGGGGACAGTCAGGCAGATAGA	3	202–206	0.52	0.54
FJ535716		GCAGCAGATGCCTTTAACATTAGG				
*LcaE100*	(TG)_7_	CGCGGGTGTACACTTTGTATA	2	64–74	0.45	0.33
FJ535717		CTCAGTGTTTCTTTCTCAATGTCA				
*LcaE102*	(TG)_10_	CGCGGGGTTTAAGGAATGTAAG	3	176–180	0.45	0.38
FJ535718		AGGAAGGCCACGGTGATGG				
*LcaE108*	(TG)_7_	GAGCAGCTGGCCGGAAAGTAAAT	2	172–178	0.38	0.25
FJ535719		AGGCAGCAGAGGGAAAAGAAAATA				
*LcaE111*	(CA)_7_	AGTATACATGCCAGCAGTGTTACA	2	180–192	0.33	0.33
FJ535720		ATTTTTCCATCCTCGCTTCTC				
*LcaE118*	(CA)_9_	GGCCACGACGCGTAATCACTC	3	102–110	0.25	0.46*
FJ535721		TGCCCTGCTTCTTTTCCCTTCT				
*LcaE120*	(TC)_9_	ACCTCCCTCCAGACCATAAACAAC	3	144–160	0.33	0.35
FJ535722		AAGTCAGCCACGCTATACGCTTAG				
*LcaE124*	(TG)_15_	GGAAACAGGCTCCATCATCTTA	2	166–170	0.46	0.40
FJ535723		CACAACCGCTAAAGGCTATCA				
*LcaE12*5	(TG)_8_	AAGCAAAGCAAAGCTCGGCTCTAT	5	212–220	0.63	0.76
FJ535724		TGAAGGTGCTTGGCGTCAGAT				
LcaE187	(TC)_14_	AAGCGCCTTTCTGTTCGTTATTC	10	146–178	0.79	0.86
FJ535725		TGAGGGGATCAGAAATGAAAACTC				
*LcaE212*	(GA)_17_	TGAGGCATTTCTGAGAACCAACAC	7	264–276	0.59	0.77*
FJ535726		AGGCTGGGCCAGATAAACACAA				
*LcaE218*	(TG)_12_	AACAAATGTGGCAAAAAGGTCTGC	4	280–288	0.13	0.20*
FJ535727		TTTGGGAAACTGCTCAACATCACC				
*LcaE223*	(CA)_10_	GACCGCCCCTTCCTCTACCTGAT	7	156–164	0.70	0.76
FJ535728		CAAACGCAAAGCAAACACCTGAAA				
*LcaE241*	(CA)_16_	GGAGGCCATGGAGAAGCAGATAG	6	199–217	0.50	0.72
FJ535729		CCTTTGGGCAGTACTGTATTTTTC				
*LcaE246*	(TG)_7_	CGCGGGTGAGACTATTACAGA	3	276–280	0.44	0.42
FJ535730		CAGTGCTGCGATATGTCTATTCAT				
*LcaE248*	(TG)_12_	CTCGGCCAGGTCTGATGAGT	8	169–187	0.83	0.46*
FJ535731		TAATACTGACGTCGCCTCGTTC				
*LcaE250*	(TG)_7_	GGCTCTCCCGTCTCCAGGTTT	3	94–104	0.42	0.51*
FJ535732		CTGCGCCCTCCCATCAGTG				

*H*_*O*_, observed heterozygosity; *He*, expected heterozygosity; *HWE, *P *< 0.05

## Conclusion

We have developed a very simple and highly efficient method for identifying microsatellites from cDNA. Microsatellites isolated from cDNA showed polymorphism and a Mendelian pattern of inheritance. Therefore, the method will be ideal for isolation of microsatellites from cDNA of fish species where there are no EST sequences available or the number of ESTs is limited.

## Methods

### Identification of microsatellites from existing ESTs of Asian seabass (Lates calcarifer)

In a previous study we sequenced 4800 ESTs from six normalized cDNA libraries of Asia seabass [10]. Microsatellites in these ESTs were identified using SciRoKo 3.1 [23]. Default parameters were used in the search for microsatellites. SciRoKo provides statistical analysis of the microsatellites.

### Isolating microsatellites from cDNA

#### Synthesis of first strand cDNA and second cDNA

Total RNA was isolated from brain of a 3-months old Asian seabass using Trizol (Invitrogen) according to the manufacturer's protocol. DNA residue in the RNA was removed with the treatment of DNAse (NEB). First strand DNA was synthesized using CDS primer [AAGCAGTGTATCAACGCAGAGTA(T35)], Smart oligo II A primer (AAGCAGTGTATCAACGCAGAGTACrGrGrG) and PowerScript reverse transcriptase (BD Bioscience) according to BD Bioscience's protocol. The reaction consisted of 3 μg total RNA, 1 mM CDS primer, and 1 mM smart oligo II A primer, 1 × first strand buffer (BD Bioscience), 2 mM DTT and 1 mM of each dNTP in 10 μl. The reaction was incubated at 42°C for 2 hours on a PTC-100 PCR machine (MJ research) and then cooled on ice.

Second strand cDNA was synthesized using SMART cDNA technology. Briefly, the synthesized first strand cDNA was 1: 6 diluted in 1 × TE (pH 8.0), heated at 72°C for 10 min, and used in the synthesis of the second strand cDNA according to BD Bioscience's protocol. The 50 μl reaction comprised of 1 μl diluted first strand cDNA, 1 × buffer (BD Bioscience), five units of polymerase mix (BD Bioscience), 200 μM dNTPs, 0.25 μM smart PCR primer (AAGCAGTGTATCAACGCAGAGT). PCR was performed on PTC-100 PCR machine using the following program: 20 cycles of 95°C for 8 s, 68°C for 20 s and 72°C for 3 min.

#### Normalization of smart cDNA

Normalization of cDNA was conducted using DSN (duplex-specific nuclease) (Evrogen) [20] according to the manufacturer's recommendation. Briefly, the amplified second strand cDNA was cleaned using glassmilk (Gen101) and diluted to 50 ng/μl. Three microliter of cDNA with 1 μl 4× hybridization buffer [200 mM Hepes-HCl (pH 8.0), 2 M NaCl] was denatured at 95°C for 5 min, and then incubated at 68°C for 4 h for renaturation. After the incubation, the following reagents preheated at 68°C were added to the hybridization reaction: 3 μl water, 1 μl 5 × DSN buffer [500 mM Tris-HCl (pH 8.0); 50 mM MgCl_2 _and 10 mM DTT] and 0.5 μl (1 U/μl) DSN (Evrogen). The 10 μl reaction was incubated at 65°C for 30 min on a PTC-100 PCR machine followed by heating at 95°C for 8 min to inactivate the DSN. The normalized cDNA was diluted 4 times with water, and amplified with the Smart PCR primer for 20 cycles as described in the above section.

#### Incorporating Sal I and Not I linkers to the ends of cDNA

To produce directional microsatellite-enriched cDNA libraries, the 5' and 3' ends of the normalized cDNA were annealed a linker with a cutting site *Sal I *and *Not I *respectively by the following 50 μl PCR reaction: 1 μl (20 times diluted) smart cDNA or normalized smart cDNA, 1 × advantage 2 buffer (BD Bioscience), 200 μM dNTPs, 1 μl (5 units) advantage 2 polymerase mix (BD Bioscience), 0.15 μM NotI-T25 primer [AATGTCGAGVGGCCGCGTAC(T)25], 0.15 μM SalI primer (TTGTAGCGTCGACTCACTATC), 0.015 μM SalI smart primer (TTGTAGCGTCGACTCACTATCAAGCAGTGTATCAACGCAGA). The PCR was performed on a PTC-100 PCR machine with the following program: 20 cycles of 95°C for 8 s, 68°C for 20 s and 72°C for 3 min.

#### Enrichment of microsatellites

Microsatellites in cDNA were enriched by using biotinylated oligonucleotides and streptavidin-coated magnetic beads. Briefly, 1 μg cDNA in 6 × SSC was denatured at 98°C for 5 min, followed by hybridization with 1 μl 10 pmol/μl biotinylated (CA)_10 _or (GA)_10 _in 65 μl 6 × SSC at 55°C for 25 min. DNA hybridization products (65 μl) were captured with 35 μl (ca. 350 μg) streptavidin coated beads (Pierce) (suspended in 6 × SSC) which were washed twice in 1 × TE (pH 8.0) and twice in 6 × SCC before capture at room temperature. Beads capturing microsatellite-enriched cDNA were washed twice in 2 × SSC containing 0.1% SDS and twice in 1 × SSC at room temperature, and then a final wash in 1 × SSC at 55°C for five min. The captured cDNA was eluted with 30 μl water and PCR-amplified in a reaction of 25 μl consisted of 3 μl eluted cDNA, 200 nM SalI primer, 200 nM NotI-T25 primer, 200 μM dNTPs, 1 × PCR buffer, and two units of polymerase mix (BD Bioscience). The PCR was carried out on a PTC-100 PCR machine using the following program: 30 cycles of 95°C for 8 s, 65°C for 20 s and 72°C for 3 min. PCR products were cleaned and concentrated using glassmilk (Gen 101).

#### Directional cloning of microsatellite-enriched cDNA

The microsatellite-enriched cDNA PCR products was digested with S*alI *and *NotI *using the following protocol: 20 μl (ca. 500 ng) cleaned cDNA, 3 μl 10 × S*alI *buffer, 1 μl (20 units) *SalI *(NEB) and 1 μl (10 units) *NotI *(NEB) at 37°C for 2 hours. After digestion, the cDNA was electrophoresed on 1% low melt gel (BIO-RAD). Fragments between 500 bp and 1200 bp were excised and cleaned using glassmilk (Gen 101). Approximately 50 ng cDNA was ligated to 25 ng pCMV-SPORT-6 vector (Invitrogen) which was used to transform XL-blue supercompetent cells (Stratagene). Schematic presentation of the method for microsatellite enrichment from normalized cDNA is shown in Figure 1.

#### Sequencing of clones

White colonies were picked and arrayed into 96-well plates containing 40 μl LB liquid medium with 100 μg/ml ampicillin in each well. The 96 well plates were cultured at 37°C for 16–18 hours without shaking. Inserts of each colony were PCR amplified using two microliter cell culture in LB as template, low concentration (50 nM) of M13PUC forward (5' CCCAGTCACGACGTTGTAAAACG 3') and reverse primers (5' AGCGGATAA-CAATTTCACACAGG 3') with the following PCR program: 94°C for 5 min followed by 35 cycles of 94°C at 30 s, 55°C for 30 s and 72°C for 48 s, with a final extension at 72°C for 5 min. Two microliters of colony PCR products were directly sequenced in both directions using M13PUC-F/M13PUC-R primers and BigDye kit on an ABI3730xl sequencer (both from Applied Biosystems). Forward and reverse sequences were assembled using software Sequencher (GeneCodes). Primers were designed for a subset of microsatellites in the flanking regions using PrimerSelect (Dnastar). One primers of each pair was labeled with a fluorescent dye Hex or Fam (1^st ^Base). DNA sequences of the polymophic microsatellites were deposited in GenBank with the accession numbers: EF210110–EF210125 and FJ535708–FJ535732.

#### Characterization of microsatellites isolated from normalized cDNA

PCR amplification of microsatellites was performed on a PTC-100 thermal cycler in a 25 μl reaction volume containing 100 ng DNA, 1 × PCR buffer [50 mM KCl, 10 mM Tris-HCl (pH 8.8), 1.5 mM MgCl_2 _and 0.1% Triton-X 100], 200 nM of each primer, 50 μM of each dNTP and one unit DNA polymerase (Finnzymes). Cycling conditions were: 94°C for 2 min followed by 35 cycles of 94°C for 30 s, 55°C for 30 s and 72°C for 30 s, with a final extension at 72°C for 5 min. Fluorescence-based genotyping of 24 unrelated Asian seabass individuals originated from Southeast Asia and Australia was conducted using an automated DNA sequencer ABI 3730xl (Applied Biosystems). Each microsatellite was examined for genotyping errors using MicroChecker [24]. Mendelian inheritance patterns of all microsatellites were examined on one of three pedigrees, each including one parental pair and 24 offspring using the chi-square test. Hardy-Weinberg Equilibrium (HWE) and linkage disequilibrium were examined using GDA [25]

## List of abbreviations

cDNA: complementary DNA; EST: expressed sequence tag; DSN: duplex-specific nuclease.

## Authors' contributions

GHY planned and started the project, drafted and determined the final version of the manuscript. ZZY, WCM and XJH conducted the experiments. All authors have read and approved the final version of the manuscript.

## References

[B1] Weber JL, May PE (1989). Abundant class of human DNA polymorphisms which can be typed using the polymerase chain reaction. Am J Hum Genet.

[B2] Tautz D (1989). Hypervariability of simple sequences as a general source for polymorphic DNA markers. Nucleic Acids Res.

[B3] Goldstein DB, Schlotterer C (1999). Microsatellites: Evolution and Applications.

[B4] Zane L, Bargelloni L, Patarnello T (2002). Strategies for microsatellite isolation: a review. Mol Ecol.

[B5] Vignal A, Milan D, SanCristobal M, Eggen A (2002). A review on SNP and other types of molecular markers and their use in animal genetics. Genet Sel Evol.

[B6] Streelman JT, Kocher TD (2002). Microsatellite variation associated with prolactin expression and growth of salt-challenged tilapia. Physiol Genomics.

[B7] Smith CT, Baker J, Park L, Seeb LW, Elfstrom C, Abe S, Seeb JE (2005). Characterization of 13 single nucleotide polymorphism markers for chum salmon. Mol Ecol Notes.

[B8] Yue GH, Ho MY, Orban L, Komen J (2004). Microsatellites within genes and ESTs of common carp and their applicability in silver crucian carp. Aquaculture.

[B9] Liu Z, Tan G, Li P, Dunham RA (1999). Transcribed dinucleotide microsatellites and their associated genes from channel catfish *Ictalurus punctatus*. Biochem Biophys Res Commun.

[B10] Xu YX, Zhu ZY, Lo LC, Wang CM, Lin D, Feng F, Yue GH (2006). Characterization of two parvalbumin genes in Asian seabass (*Lates calcarifer*). Anim Genet.

[B11] Ju Z, Wells MC, Martinez A, Hazlewood L, Walter RB (2005). An in silico mining for simple sequence repeats from expressed sequence tags of zebrafish, medaka, *Fundulus*, and *Xiphophorus*. In Silico Biol.

[B12] Wang HX, Li FH, Xiang JH (2005). Polymorphic EST-SSR markers and their mode of inheritance in *Fenneropenaeus chinensis*. Aquaculture.

[B13] Quilang J, Wang S, Li P, Abernathy J, Peatman E, Wang Y, Wang L, Shi Y, Wallace R, Guo X (2007). Generation and analysis of ESTs from the eastern oyster, *Crassostrea virginica Gmelin *and identification of microsatellite and SNP markers. BMC Genomics.

[B14] Slate J, Hale MC, Birkhead TR (2007). Simple sequence repeats in zebra finch (*Taeniopygia guttata*) expressed sequence tags: a new resource for evolutionary genetic studies of passerines. BMC Genomics.

[B15] Palmieri DA, Novelli VA, Bastianel M, Cristofani-Yaly M, Astúa-Monge G, EF C, de Oliveira AC, Machado MA (2007). Frequency and distribution of microsatellites from ESTs of citrus. Genet Mol Biol.

[B16] Luro FL, Costantino G, Terol J, Argout X, Allario T, Wincker P, Talon M, Ollitrault P, Morillon R (2008). Transferability of the EST-SSRs developed on *Nules clementine *to other *Citrus *species and their effectiveness for genetic mapping. BMC Genomics.

[B17] (2008). Fishbase. http://www.fishbase.org/.

[B18] Nonneman D, Waldbieser GC (2005). Isolation and enrichment of abundant microsatellites from a channel catfish (*Ictalurus punctatus*) brain cDNA library. Anim Biotechnol.

[B19] Yue GH, Chen F, Orban L (2000). Rapid isolation and characterization of microsatellites from the genome of Asian arowana (*Scleropages formosus, Osteoglossidae, Pisces*). Mol Ecol.

[B20] Zhulidov PA, Bogdanova EA, Shcheglov AS, Vagner LL, Khaspekov GL, Kozhemyako VB, Matz MV, Meleshkevitch E, Moroz LL, Lukyanov SA (2004). Simple cDNA normalization using kamchatka crab duplex-specific nuclease. Nucleic Acids Res.

[B21] Hirozane-Kishikawa T, Shiraki T, Waki K, Nakamura M, Arakawa T, Kawai J, Fagiolini M, Hensch TK, Hayashizaki Y, Carninci P (2003). Subtraction of captrapped full-length cDNA libraries to select rare transcripts. Biotechniques.

[B22] Zhu ZY, Wang CM, Lo LC, Feng F, Lin G, Yue GH (2006). Isolation, characterization, and linkage analyses of 74 novel microsatellites in Barramundi (*Lates calcarifer*). Genome.

[B23] Kofler R, Schlötterer C, Lelley T (2007). SciRoKo: A new tool for whole genome microsatellite search and investigation. Bioinformatics.

[B24] Oosterhout CV, Hutchinson MF, Wills DPM, Shipley P (2004). MICROCHECKER: software for identifying and correcting genotyping errors in microsatellite data. Mol Ecol Notes.

[B25] Lewis PO, Zaykin D Genetic Data Analysis. http://hydrodictyon.eeb.uconn.edu/people/plewis/software.php.

